# Individual Differences in the Effect of Orthographic/Phonological Conflict on Rhyme and Spelling Decisions

**DOI:** 10.1371/journal.pone.0119734

**Published:** 2015-03-09

**Authors:** Suzanne E. Welcome, Amanda C. Alton

**Affiliations:** Department of Psychology, University of Missouri—St. Louis, St. Louis, Missouri, United States of America; The National Institutes of Health, UNITED STATES

## Abstract

In typical readers, orthographic knowledge has been shown to influence phonological decisions. In the present study, we used visual rhyme and spelling tasks to investigate the interaction of orthographic and phonological information in adults with varying reading skill. Word pairs that shared both orthography and phonology (e.g., *throat/boat*), differed in both orthography and phonology (e.g., *snow/arm*), shared only orthography (e.g., *farm/warm*), and shared only phonology (e.g., *vote/boat*) were visually presented to university students who varied in reading ability. For rhyme judgment, participants were slower and less accurate to accept rhyming pairs when words were spelled differently and to reject non-rhyming pairs when words were spelled similarly. Similarly, for spelling judgments, participants were slower and less accurate when indicating that word endings were spelled differently when words rhymed, and slower and less accurate when indicating that words were spelled similarly when words did not rhyme. Crucially, while these effects were clear at the group level, there were large individual differences in the extent to which participants were impacted by conflict. In two separate samples, reading skill was associated with the extent to which orthographic conflict impacted rhyme decisions such that individuals with better nonword reading performance were less impacted by orthographic conflict. Thus, university students with poorer reading skills may differ from their peers either in the reading strategies they use or in the degree to which they automatically access word form information. Understanding these relationships is important for understanding the roles that reading processes play in readers of different skill.

## Introduction

In skilled readers, connections have been forged between a phonological (sound-based) system and an orthographic (visually-based) system. These connections may mean that literacy fundamentally changes the way in which language is processed. A number of studies have demonstrated that in skilled readers, orthographic information influences phonological decisions and recognition of spoken words e.g. [1, 2]. A classic finding is that the speed and accuracy of rhyme judgments can be impacted by word spelling, such that adult participants are faster to recognize that similarly spelled pairs (e.g. *tie-pie)* rhyme than rhymes that are spelled differently (e.g., *rye-tie)* [1]. This increase in response latency for pairs in which orthographic and phonological information conflict (incongruent word pairs) over congruent pairs has been demonstrated using both auditory and visual presentation of word pairs [3, 4].

Other paradigms have also been used to demonstrate that orthographic information influences auditory word processing. For example, Ziegler and Ferrand [2] demonstrated that auditory lexical decision times are impacted by consistency, such that inconsistent words, whose rimes can be spelled multiple ways (e.g., *leap)*, were recognized more slowly than consistent words, whose rimes can only be spelled one way (e.g., *duck*). This effect of consistency extends to semantic and gender categorization tasks, indicating that orthography influences many aspects of spoken word processing [5]. In a priming paradigm, primes that shared both orthography and phonology with a target were more effective primes than those that shared phonology only [6]. During a phoneme monitoring task, adults were faster to respond to a target phoneme when it was spelled typically than when it was spelled atypically [7]. Words with more orthographic neighbors show faster reaction times in a shadowing task [8].

There is mixed evidence regarding interactions between orthography and phonology in individuals with poor phonological decoding skills. Some evidence supports the view that orthographic processing is preserved in those with difficulties in phonological decoding. In individuals with dyslexia, orthographic processing is impacted less severely and persistently than phonological processing [9]. As poor readers mature, their deficit in orthographic processing is reduced while their phonological deficit is increased (relative to normal-reading peers), suggesting that individuals with dyslexia are able to catch up in orthographic processing while falling behind in phonological processing [10]. Proficient and dyslexic adult readers showed a comparable effect of orthography on auditory rhyme judgments [11], suggesting that individuals with adequate reading experience, regardless of reading skill, show similar influence of orthography when the task is metaphonological in nature, requiring explicit identification and analysis of speech sounds.

Other findings suggest that individuals with poor phonological processing skills exhibit reduced access to orthographic information. Children with dyslexia showed less orthographic facilitation than typical reading groups in an auditory rhyme detection task [12]. Children with dyslexia also did not show a typical orthographic neighborhood effect [13], showing little benefit for the processing of words that share orthography with many other words. In an fMRI study, children with reading disabilities failed to show reliable activation of the fusiform cortex during an auditory rhyming task [14]. The fusiform gyrus typically shows activation during visual or orthographic processing, and activation of this region in typically-reading children was taken as evidence for activation of orthographic information during an auditory task.

However, there are some reports that individuals with poor phonological skills show enhanced, rather than reduced, effects of orthography. Some individuals with impaired phonological decoding skills were shown to excel at orthographic tasks [15, 16]. Among adolescents performing a visual rhyming task, individuals with dyslexia showed high error rates when phonological and orthographic information conflicted [17]. Adult literacy students, who read at grade levels 3 to 6, made more errors than reading-level matched children on rhyming trials with different spelling [18], indicating that these participants might rely on spelling information to make rhyme decisions.

While the effect of orthography on rhyme decisions and spoken language processing is clear at a group level, it is less clear whether meaningful variation in this effect exists within the adult population. However, Dich [19] demonstrated a relationship between an individual’s spelling skill and the extent to which orthography impacts the recognition of spoken words. Individuals with stronger spelling skills tended to show larger effects of orthography on an auditory lexical decision task (a greater difference between inconsistent and consistent words). A more complicated pattern of results was found when comparing effects of orthography on phonological processing between typical readers and dyslexic adults [11]. During an auditory lexical decision task, consistency effects were related to reading skill, with better readers showing a larger effect of consistency. This relationship with reading skill was not observed for an auditory rhyme judgment task. The authors attributed this pattern of results to the metaphonological nature of the rhyme task. Together, such findings suggest that individual differences in reading and spelling ability may modulate the effect of orthography on phonological tasks.

In the present studies we used visual rhyme and spelling tasks to investigate the interaction of orthographic and phonological information in adults with varying reading skill. Prior studies have demonstrated effects of conflicting orthography on phonological judgments with both auditory and visual presentation of stimuli. Those studies have demonstrated an increase in response latency for orthographically dissimilar rhyme pairs over orthographically similar rhyme pairs using both visual and auditory word presentations [20, 21]. Event-related potential (ERP) data further suggest that such effects are not fully dependent on modality. Consistent differences between non-rhyming and rhyming targets emerge about 300 ms after stimulus onset for both auditory [22] and visual [23] word pairs. Further, ERPs have revealed effects of conflicting orthography during rhyme decisions and spoken language processing with effects of orthography emerging during the same time window as effects of rhyme, using visual presentation [20, 21]. These findings make sense in light of the fact that in order to make a rhyme judgment, the phonological form of the word must be accessed, regardless of the modality of presentation. Nonetheless, differences between modalities exist in terms of the need for print-to-sound conversion and the saliency of the task-irrelevant information.

We were particularly interested in relationships with reading skill. Within the university population, there is considerable variability in reading skill, particularly the ability to read nonwords [24, 25]. Understanding the consequences of this variability in the adult population is important, as readers with varying skill might engage different mechanisms during reading, and a full understanding of the behavioral and neural underpinnings of reading mechanisms must incorporate this variability. Specifically, adults with relatively poor phonological decoding skills may differ from other readers in orthographic processing [26, 27]. On this basis, we predicted that there would be a relationship between phonological decoding skill and the extent to which conflict between orthographic and phonological information impacted an individual such that individuals with worse nonword reading skill would be more reliant on orthographic information, and thus more sensitive to phonological conflict. Most previous studies exploring these questions primarily compared effects in dyslexic and typical-reading children. In this study, we investigated effects across the reading spectrum in adults, in whom reading processes are likely fully developed. For both rhyme and spelling tasks, it is possible for conflict to exist between orthographic and phonological information, and the use of two tasks allows us to investigate the effect of this conflict on both phonological and orthographic decisions.

## Experiment 1 Methods

### Participants

Forty-three neurologically normal adults (38 female) took part in the study and were compensated with course credit. All were right-handed native speakers of English. The study had the approval of the University of Missouri—St Louis (UMSL) Institutional Review Board and written informed consent was obtained from all participants. Seven participants were removed because their behavioral performance indicated imperfect understanding of the task (accuracy was below 60% on two or more experimental conditions). See S1 Table: Mean (standard deviation) performance across condition for seven participants dropped from Experiment 1. Note that major results are not changed by the inclusion of these participants with low accuracy (S2 Table: Associations (Pearson’s r-values) between effects of conflict and cognitive performance including all participants from Experiment 1). For the remaining 36 participants, scores on standardized measures of reading and nonverbal intelligence ranged from below average to well above average, reflecting the variability present in the UMSL student body (Table 1).

**Table 1:** Demographic characteristics of the sample for Experiment 1 (N = 36).

	Mean (SD)	Range
**Age**	22.3 (5.2)	18–42
**Adult Reading History Questionnaire**	0.29 (0.10)	0.10–0.52
**TONI (Scaled Score)**	97.8 (9.0)	85–124
**TOWRE SWE (Scaled Score)**	97.4 (10.8)	76–130
**TOWRE PDE (Scaled Score)**	100.8 (11.5)	67–127

### Psychometric Measures

All participants completed standardized measures of intelligence and reading ability. Nonverbal intelligence was assessed with the Test of Nonverbal Intelligence-3, which uses picture stimuli to evaluate skill at recognizing logical sequences (TONI-3) [28]. The Sight Word Efficiency (SWE) and Phonemic Decoding Efficiency (PDE) subtests of the Test of Word Reading Efficiency (TOWRE) were used to assess timed reading of familiar words and pronounceable pseudowords, respectively [29]. Participants completed the Adult Reading History Questionnaire (ARHQ) [30], designed to investigate participants’ childhood reading experiences and current attitudes. This questionnaire is designed to provide a cut-score (0.30) above which scores indicate a history of reading disability. Using this cut-score, 17 participants would be classified as having a positive history of reading disability. UMSL’s undergraduate student body is somewhat nontraditional, with more than 50% transfer students and a mean age of 26, and it might therefore not be surprising that a relatively large portion of the sample experienced reading difficulty at some point in the past. Despite the relatively high scores on this measure, none of the participants reported repeating grades due to academic failure. Some items in the questionnaire did not seem applicable to the college population (e.g., reading of newspapers given that many of the participants report seeking news through other media), so we did not use a cut-score, and instead treated the score as a continuous measure.

There was a significant association between performance on the SWE and PDE subtests of the TOWRE, *r*(34) = .48, *p* < .005. However, nonverbal intelligence and scores on the ARHQ were not significantly correlated with each other, *r*(34) = −.19, *p* > .10, or with either reading measure [TONI with SWE: *r*(34) = −.02, *p* > .10; TONI with PDE: *r*(34) = −.04, *p* > .10; ARHQ with SWE: *r*(34) = .20, *p* > .10; ARHQ with PDE: *r*(34) = −.20, *p* > .10]. One explanation for the lack of significant associations between self-reported reading history and current reading skill is that the ARHQ contains a mix of questions relating to current reading behavior and past reading experiences. It is possible that participants obtained high scores on the self-report measure primarily as a result of academic difficulty during childhood, and these early experiences are only weakly related to adult performance.

### Materials

All participants made rhyme and spelling decisions on visually presented word pairs. In total, participants saw 80 pairs sharing both orthography and phonology (O+P+ pairs), 80 pairs sharing neither orthography nor phonology (O-P- pairs), 40 pairs sharing phonology but not orthography (O-P+ pairs), and 40 pairs sharing orthography but not phonology (O+P- pairs). Three pairs from each of 40 O-P-, O+P+, and O-P+ triplets (seek/boat, throat/boat, vote/boat) and 40 O-P-, O+P+, O+P- triplets (snow/arm, farm/arm, warm/arm) were presented to participants. In this manner, targets were preceded by words that varied in their relationship to the target. Length, concreteness [31], familiarity [31], log frequency [32], number of orthographic neighbors [33], and bigram frequency [33] were equated as much as possible for target words, orthographically related words, and orthographically unrelated words [*ts* (78) < 1.5, *p*s > .10] across conditions.

### Procedure

Participants performed both a visual rhyme decision task and a visual spelling task, with task order counterbalanced across participants. All stimuli were presented in lowercase, black 18 point Arial font on a white background. Each trial began with a fixation cross that flickered for 250 ms. On each trial, a visual word was presented for 500 ms, followed by an ISI of 500 ms, and presentation of a target word for 500 ms. Trials were separated by a 1,000 ms inter-trial interval. Participants indicated their decision with a button press. For the rhyme task, participants were instructed to indicate their rhyme/no rhyme decision with a button press. For the spelling task, participants were asked to indicate whether the endings of the word pair were spelled similarly with a button press. Participants used their index finger on the “1” key and their middle finger on the “2” key to indicate their response, with response mapping counterbalanced across participants. Before each task, 12 practice trials were administered. Each task was broken into 4 blocks of trials that lasted approximately 3–4 minutes.

## Experiment 1 Results

### Overall Effects

A 2 (task) x 2 (phonology) x 2 (orthography) ANOVA was conducted separately for accuracy and RT. For accuracy, a main effect of task was observed, *F*(1, 35) = 4.17, *p* < .05, with better performance on the rhyme task. The main effects of phonology and orthography were not significant (*F*s < 3). The interaction of task and phonology was significant, *F*(1, 35) = 33.7, *p* < .001. For the rhyming task, shared phonology led to higher accuracy (90.2% versus 85.9%), while for the spelling task, shared phonology led to less accuracy (81.4% versus 89.1%). The interaction of task and orthography was also significant, *F*(1, 35) = 26.2, *p* < .001. For the rhyming task, shared orthography led to less accuracy than non-shared (85.5% versus 90.6%), while for the spelling task, shared orthography led to more accuracy than non-shared (88.0% versus 82.5%). Thus, accuracy was higher for the rhyme task when items shared phonology and higher for the spelling task was items shared orthography. Crucially, phonology and orthography showed a significant interaction, *F*(1, 35) = 80.92, *p* < .001. When phonology was shared, shared orthography led to more accuracy (91.3% versus 80.3%). When phonology differed, shared orthography led to less accuracy (82.2% versus 92.8%). The task x phonology x orthography interaction was not significant (*F* < 1).

For RT, a main effect of task was observed, *F*(1, 35) = 25.8, *p* < .001, with faster responses to the spelling task. The main effect of phonology was not significant (*F* < 1.6), but the main effect of orthography was, *F*(1, 35) = 7.4, *p* < .01. The interaction of task and phonology was significant, *F*(1, 35) = 96.6, *p* < .001. For the rhyming task, shared phonology led to lower RTs (704 versus 760), while for the spelling task, shared phonology led to longer RTs (688 versus 648). The interaction of task and orthography was also significant, *F*(1, 35) = 89.3, *p* < .001. For the rhyming task, shared orthography led to longer RTs than non-shared (761 versus 703), while for the spelling task, shared orthography led to shorter RTs than non-shared (650 versus 685). Thus, performance was better for the rhyme task when items shared phonology and better for the spelling task was items shared orthography. Crucially, phonology and orthography showed a significant interaction, *F*(1, 35) = 238.3, *p* < .001. When phonology was shared, shared orthography led to faster RTs than differing orthography (649 versus 744). When phonology differed, shared orthography led to longer RTs (762 versus 646). The task x phonology x orthography interaction was not significant (*F* < 2.5).

Performance across conditions is presented in Table 2. Planned comparisons using paired-samples t-tests indicated that accuracy was higher for O+P+ trials than O-P+ trials [*t*(35) = 3.19, *p* < .005] and higher for O-P- trials than O+P- trials [*t*(35) = 6.25, *p* < .001]. RTs also significantly varied with condition [*F*(3, 77.8) = 102.6, *p* < .001]. RTs were shorter for O+P+ trials than O-P+ trials [*t*(35) = 7.23, *p* < .001], and shorter for O-P- trials than O+P- trials [*t*(35) = 15.88, *p* < .001]. Thus, orthographic overlap within a trial led to faster and more accurate rhyme decisions and slower and less accurate no-rhyme decisions.

**Table 2:** Mean (standard deviation) performance across condition from Experiment 1. Accuracies are percent correct and RTs are in ms.

		O+P+	O-P-	O-P+	O+P-
**Rhyming**	Accuracy	92.7 (4.2)	93.5 (4.1)	87.7 (11.1)	78.4 (15.8)
	RT	677 (113)	675 (111)	732 (125)	845 (118)
**Spelling**	Accuracy	89.9 (6.3)	92.1 (5.5)	72.9 (16.8)	86.0 (7.4)
	RT	621 (115)	616 (113)	755 (149)	679 (141)

For the spelling task, a repeated measures ANOVA with Greenhouse-Geisser correction showed that accuracy significantly varied with condition (Table 2) [*F*(3, 53.1) = 31.9, *p* < .001]. Planned comparisons using paired-samples t-tests indicated that accuracy was higher for O+P+ trials than O+P- trials [*t*(35) = 5.80, *p* < .001] and higher for O-P- trials than O-P+ trials [*t*(35) = 4.25, *p* < .001]. RTs also significantly varied with condition [*F*(3, 88.2) = 78.41, *p* < .001]. RTs were shorter for O+P+ trials than O+P- trials [*t*(35) = 6.14 *p* < .001], and shorter for O-P- trials than O-P+ trials [*t*(35) = 10.79, *p* < .001]. Thus, phonological overlap within a trial led to better performance when words were spelled similarly worse performance when words were spelled differently.

To quantify the effects of orthographic/phonological conflict for each individual, differences between the average performance on the trials without conflict (O+P+ and O-P-) and trials with conflict (O+P- and O-P+) were computed separately for each task and dependent variable. For this particular study, classifying trials as “non-conflicting” and “conflicting” has the advantage of grouping trials identically for the rhyme and spelling tasks. As shown in Table 3, there were large individual differences in the extent to which individuals showed the effects of conflict. While all group means significantly differed from 0 (Table 3), some individuals showed reversed effects in accuracy and/or small effects in RT, while others showed robust effects of conflict.

**Table 3:** Differences indicating effect of orthographic conflict on rhyming decisions and phonological conflict on spelling decisions from Experiment 1.

		Mean (SD)	Comparison of mean to 0	Range
**Rhyming**	Accuracy (difference in percent correct)	10.1 (8.9)	*t*(35) = 6.78, *p* < .001	−3.0 to 10.1
	RT (difference in ms)	113 (48)	*t*(35) = 14.1, *p* < .001	41 to 250
**Spelling**	Accuracy (difference in percent correct)	11.6 (11.0)	*t*(35) = 6.3, *p* < .001	−6.2 to 52.5
	RT (difference in ms)	99 (51)	*t*(35) = 11.6, *p* < .001	15 to 267

### Relationships with Reading Skill

Given that individuals differed in the extent to which orthographic conflict impacted rhyme decisions and phonological conflict impacted spelling decisions, we explored relationships between those individual differences and performance on the standardized measures of nonverbal IQ and reading. Significant relationships emerged between effects of orthographic conflict on rhyme decisions and Phonemic Decoding Efficiency scores (PDE; Table 4). Specifically, those individuals with better phonological decoding skills were less impacted by orthographic conflict. Interestingly, this relationship was not apparent with Sight Word Efficiency or nonverbal IQ. Additionally, no significant relationships between standardized test performance and spelling effects emerged, suggesting that reading skill relates specifically to the effect of orthographic overlap on phonological judgments, not to effects of conflict more broadly.

**Table 4:** Associations (Pearson’s r-values) between effects of conflict and cognitive performance in Experiment 1.

	Rhyme Accuracy Effect	Rhyme RT Effect	Spelling Accuracy Effect	Spelling RT Effect
**TONI (Scaled Score)**	−.06	.05	−.24	.04
**TOWRE SWE (Scaled Score)**	−.10	−.23	−.21	−.06
**TOWRE PDE (Scaled Score)**	−.36*	−.43**	−.14	−.06
**ARHQ**	.18	.10	−.09	−.18

* *p* < .05

** *p* < .01

Asterisks indicate relationships which are statistically significant.

**Fig 1:**
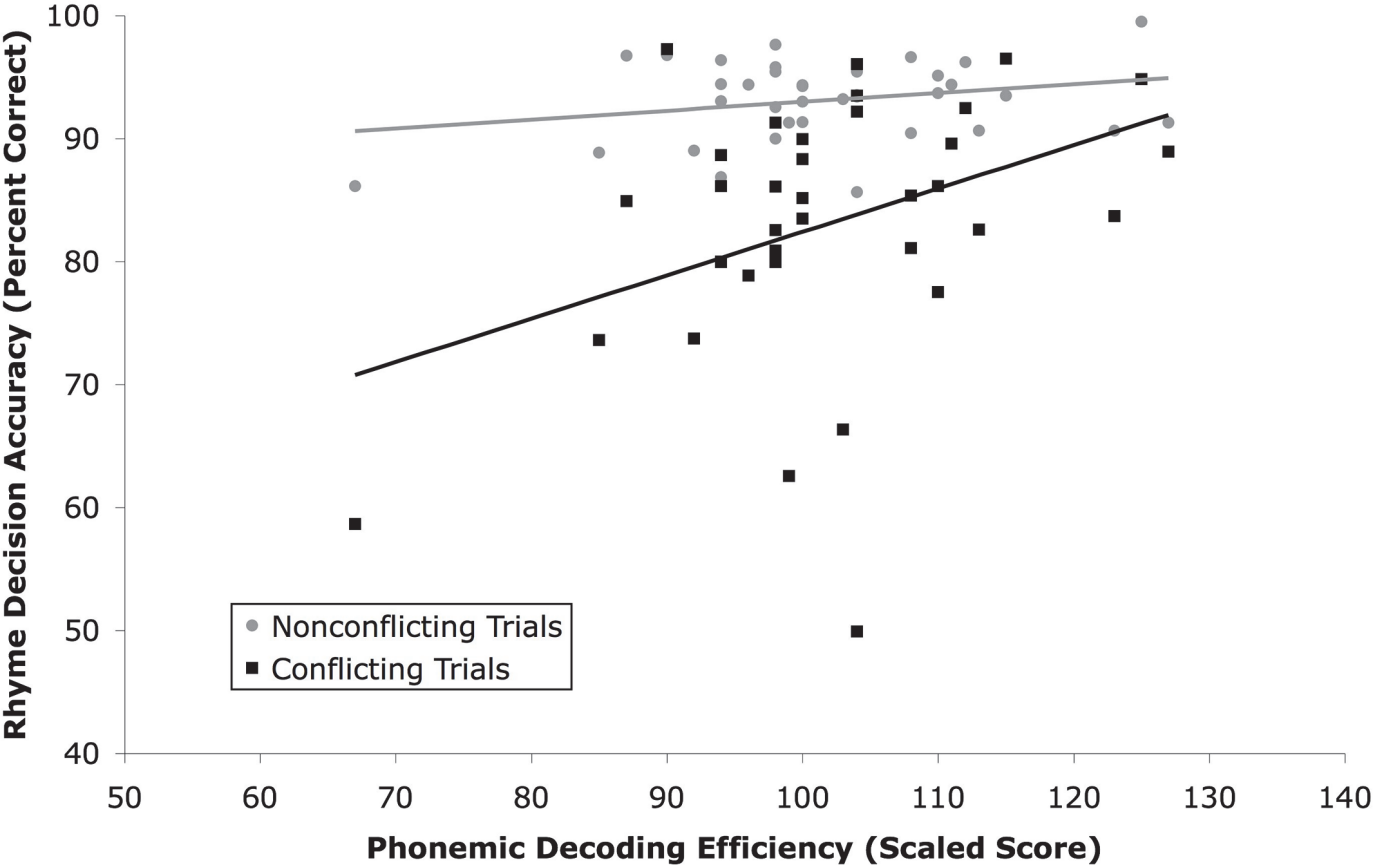
Accuracy (percent correct) of rhyme decisions made on nonconflicting (O+P+ and O-P-) trials and conflicting (O-P+ and O+P-) trials in Experiment 1.

**Fig 2:**
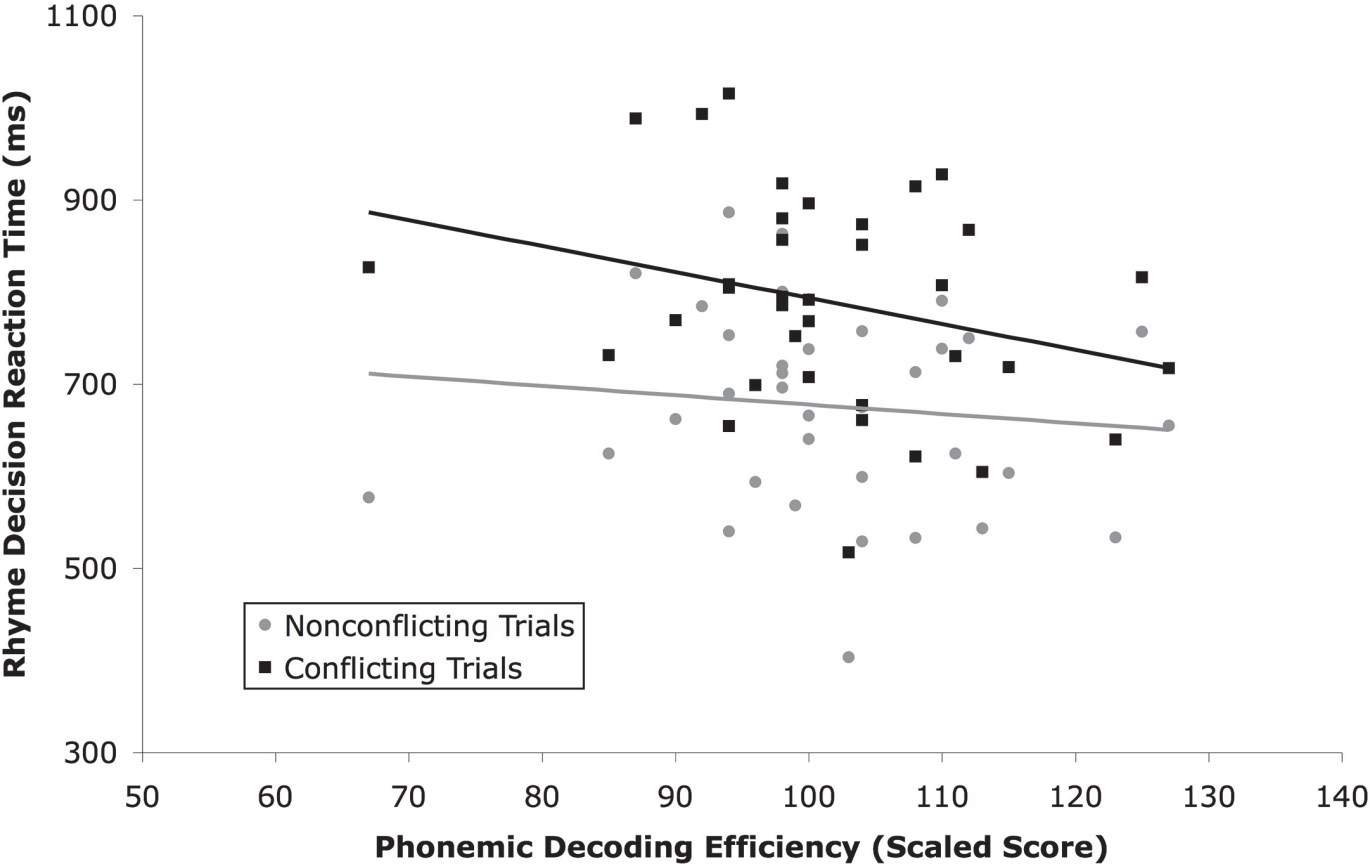
Reaction time for rhyme decisions for nonconflicting (O+P+ and O-P-) trials and conflicting (O-P+ and O+P-) trials in Experiment 1.

Relationships between PDE score and performance on each type of trial for the rhyme task were explored. PDE scores were not significantly correlated with accuracy for non-conflicting trials, *r*(34) = .25, *p* > .10, but were correlated with accuracy for conflict trials, *r*(34) = .38, *p* < .05 (Fig. 1) Accuracy was high in the non-conflict condition, regardless of PDE score. However, accuracy in the conflict condition was lower in those with lower PDE scores. A similar pattern was seen in RT (Fig. 2), though relationships failed to reach significance [non-conflict: *r*(34) = .11, *p* > .10; conflict: *r*(34) = −.28, *p* < .10]. Four participants had scaled scores on the standardized measure of reading less than 85. With those participants excluded, the association between PDE and non-conflicting trials approached significance, *r*(30) = .32, *p* < .10, while the association between PDE and conflicting trials did not, *r*(30) = .18, *p* > .10.

## Experiment 1 Discussion

Clear effects of orthography were observed for a rhyme decision task, in keeping with earlier findings that spelling can influence phonological judgments [1, 2, 6]. Accuracy was lower and RTs longer for rhyming pairs spelled differently and non-rhyming pairs that shared spelled. Similarly, conflicts between orthography and phonology impacted a spelling judgment task. Accuracy was higher and RTs shorter for word endings spelled similarly when words rhymed and for word endings spelled differently when words did not rhyme. Individuals showed this effect to different degrees, with some participants showing much larger effects of conflict than others. One predictor of the extent to which orthographic conflict impacted rhyme decisions was phonological decoding ability. Participants with worse nonword reading performance were less accurate in making phonological decisions in the face of orthographic conflict, indicating that these individuals might rely more heavily on orthographic information or experience more difficulty in inhibiting orthographic processing.

Performing the rhyme and spelling tasks within the same experimental session could have influenced results, directing participants’ attention to the spelling of word pairs. In particular, this may have exaggerated the effect of orthographic conflict on rhyme decisions. While task order was counterbalanced, half of the participants in the sample performed the spelling task first, and it is possible that those participants showed particularly large effects, coloring the results of the experiment (S1 Appendix: Results from Experiment 1 participants who performed the rhyme task first). To further address this possibility, a second group of participants was recruited to perform the rhyme and spelling tasks separately. This second group of participants also completed a measure of reading comprehension to examine relationships between orthographic/phonological interaction and comprehension skill.

## Experiment 2 Methods

### Participants

38 neurologically normal adults (28 female) that did not participate in Experiment 1 took part in the study and were compensated with partial course credit. All were right-handed native speakers of English. The study had the approval of the University of Missouri—St Louis Institutional Review Board and informed consent was obtained from all participants. Six participants were removed because their behavioral performance indicated imperfect understanding of the task (accuracy was below 60% on one or more experimental condition). See S3 Table: Mean (standard deviation) performance across condition for six participants dropped from Experiment 2. Note that major results are not changed by the inclusion of these participants with low accuracy (S4 Table: Associations (Pearson’s r-values) between effects of conflict and cognitive performance including all participants from Experiment 2.). For the remaining participants, scores on standardized measures of reading and nonverbal intelligence ranged from below average to well above average, similar to the group who participated in Experiment 1 (Table 5).

**Table 5:** Demographic characteristics of the sample for Experiment 2.

	Rhyming Experiment (N = 20)		Spelling Experiment (N = 18)	
	Mean (SD)	Range	Mean (SD)	Range
**Age**	25.5 (8.7)	18–49	29.3 (12.3)	18–63
**Adult Reading History Questionnaire**	.34 (.11)	.17–.54	.31 (.11)	.11–.46
**Nelson-Denny Reading Comprehension (Scaled Score)**	223.9 (22.1)	184–259	202.9 (17.5)	166–232
**TOWRE SWE (Scaled Score)**	106.0 (14.7)	84–140	105.5 (13.9)	84–130
**TOWRE PDE (Scaled Score)**	106.5 (8.1)	93–127	105.6 (7.6)	91–118

### Psychometric Measures

All participants completed standardized measures of reading ability. Form G of the Nelson-Denny Reading Comprehension subtest [34], in which comprehension questions follow short passages, was used as a timed measure of reading comprehension. As in Experiment 1, participants completed the Sight Word Efficiency and Phonemic Decoding Efficiency subtests of the Test of Word Read Efficiency [29] and the Adult Reading History Questionnaire [30].

There was a significant association between performance on the SWE and PDE subtests of the TOWRE, *r*(36) = .63, *p* < .001. SWE performance was significantly associated with ARHQ scores, *r*(36) = −.37, *p* < .05 and reading comprehension, *r*(36) = .48, *p* < .005. However, PDE scores were not significantly correlated with either ARHQ scores, *r*(36) = −.28, *p* > .05, or reading comprehension scores, *r*(36) = .26, *p* > .10.

### Materials and Procedure

Twenty participants performed a visual rhyme decision task alone and 18 participants performed a visual spelling task alone. Stimuli and presentation were otherwise identical to those used in Experiment 1

## Experiment 2 Results

### Overall Effects

A 2 (task) x 2 (phonology) x 2 (orthography) mixed-design ANOVA was conducted separately for accuracy and RT. For accuracy, the main effect of phonology and orthography were not significant (*F*s < 1.5). The interaction of task and phonology was significant, *F*(1, 36) = 32.1, *p* < .001. For the rhyming task, shared phonology led to higher accuracy than differing phonology (95.8% versus 90.1%), while for the spelling task, shared phonology led to less accuracy (89.2% versus 93.2%). The interaction of task and orthography was also significant, *F*(1, 36) = 17.3, *p* < .001. For the rhyming task, shared orthography led to less accuracy than differing orthography (90.5% versus 95.4%), while for the spelling task, shared orthography led to more accuracy (92.7% versus 89.6%). Thus, accuracy was higher for the rhyme task when items shared phonology and higher for the spelling task was items shared orthography. Crucially, phonology and orthography showed a significant interaction, *F*(1, 36) = 63.3, *p* < .001. When phonology was shared, shared orthography led to more accuracy (95.5% versus 89.5%). When phonology differed, shared orthography led to less accuracy (87.7% versus 95.5%). The task x phonology x orthography interaction was not significant (*F* < 1).

For RT, the main effect of phonology was significant *F*(1, 36) = 5.2, *p* < .05 as was the main effect of orthography, *F*(1, 36) = 9.7, *p* < .005. These main effects were tempered by significant interaction. The interaction of task and phonology was significant, *F*(1, 36) = 18.1, *p* < .001. For the rhyming task, shared phonology led to faster RTs (714 versus 763), while for the spelling task, shared phonology led to longer RTs (744 versus 729). The interaction of task and orthography was also significant, *F*(1, 36) = 18.9, *p* < .001. For the rhyming task, shared orthography led to longer RTs than non-shared (766 versus 712), while for the spelling task, shared orthography led to shorter RTs than non-shared (732 versus 741). Thus, performance was better for the rhyme task when items shared phonology and better for the spelling task was items shared orthography. Crucially, phonology and orthography showed a significant interaction, *F*(1, 36) = 178.3, *p* < .001. When phonology was shared, shared orthography led to faster RTs than differing orthography (695 versus 762). When phonology differed, shared orthography led to longer RTs (803 versus 690). The task x phonology x orthography interaction was also significant, *F*(1, 36) = 8.2, *p* < .01.

Performance across conditions is presented in Table 6. Planned comparisons using paired-samples t-tests indicated that accuracy was not significantly higher for O+P+ trials than O-P+ trials [*t*(19) = 1.6, *p* > .10] and was higher for O-P- trials than O+P- trials [*t*(19) = 5.3, *p* < .001]. RTs also significantly varied with condition [*F*(3, 33.2) = 66.3, *p* < .001]. RTs were shorter for O+P+ trials than O-P+ trials [*t*(19) = 6.8, *p* < .001], and shorter for O-P- trials than O+P- trials [*t*(19) = 13.3, *p* < .001]. Thus, orthographic overlap within a trial led to faster rhyme decisions and slower and less accurate no rhyme decisions.

**Table 6:** Mean (standard deviation) performance across condition in Experiment 2. Accuracies are percent correct and RTs are in ms.

		O+P+	O-P-	O-P+	O+P-
**Rhyming**	Accuracy	96.6 (2.6)	95.7 (3.6)	95.0 (4.3)	84.5 (8.8)
	RT	687 (93)	682 (109)	741 (95)	845 (125)
**Spelling**	Accuracy	94.4 (3.7)	95.3 (3.9)	83.9 (10.3)	91.0 (5.9)
	RT	704 (145)	698 (120)	784 (140)	760 (174)

For the spelling task, planned comparisons using paired-samples t-tests indicated that accuracy was higher for O+P+ trials than O+P- trials [*t*(17) = 2.62, *p* < .001] and higher for O-P- trials than O-P+ trials [*t*(17) = 5.17, *p* < .001]. RTs also significantly varied with condition [*F*(3, 39.1) = 14.1, *p* < .001]. RTs were shorter for O+P+ trials than O+P- trials [*t*(17) = 5.71, *p* < .001], and shorter for O-P- trials than O-P+ trials [*t*(17) = 4.69, *p* < .001]. Thus, phonological overlap within a trial led to faster and more accurate decisions when words were spelled similarly and slower and less accurate decisions when words were spelled differently.

As in Experiment 1, differences between the average performance on the trials without conflict (O+P+ and O-P-) and trials with conflict (O+P- and O-P+) were computed separately for each task and dependent variable. As shown in Table 7, large individual difference in the extent to which individuals showed the effects of conflict were present in this sample as well.

**Table 7:** Differences indicating effect of orthographic conflict on rhyming decisions and phonological conflict on spelling decisions in Experiment 2.

		Mean (SD)	Comparison of mean to 0	Range
**Rhyming**	Accuracy (difference in percent correct)	6.4 (5.6)	*t*(19) = 5.1, *p* < .001	−8.7 to 14.8
	RT (difference in ms)	109 (31)	*t*(19) = 15.9, *p* < .001	66 to 187
**Spelling**	Accuracy (difference in percent correct)	7.4 (5.2)	*t*(17) = 6.2, *p* < .001	0.0 to 16.7
	RT (difference in ms)	70 (51)	*t*(17) = 5.9, *p* < .001	−4 to 195

### Relationships with Reading Skill

Given that individuals differed in the extent to which orthographic conflict impacted rhyme decisions and phonological conflict impacted spelling decisions, we explored relationships between those individual differences and performance on the standardized measures of nonverbal IQ and reading. Significant relationships emerged between effects of orthographic conflict on rhyme decisions and scores on the Sight Word Efficiency and Phonemic Decoding Efficiency subtests (Table 8). Specifically, those individuals with better reading skills were less impacted by orthographic conflict. Interestingly, this relationship was not apparent with reading comprehension, indicating that decoding ability specifically, not cognitive skill in general, predicted the extent to which orthography influenced the speed and accuracy of rhyme decisions. No significant relationships between standardized test performance and spelling effects emerged.

**Table 8:** Associations (Pearson’s r-values) between effects of conflict and cognitive performance in Experiment 2.

	Rhyme Accuracy Effect	Rhyme RT Effect	Spelling Accuracy Effect	Spelling RT Effect
**Nelson-Denny Comprehension (Scaled Score)**	−.23	.34	−.20	−.26
**TOWRE SWE (Scaled Score)**	−.47*	.20	.09	−.26
**TOWRE PDE (Scaled Score)**	−.52*	.33	.39	−.40
**ARHQ**	.32	−.27	.19	.19

* *p* < .05

Asterisks indicate relationships which are statistically significant.

**Fig 3:**
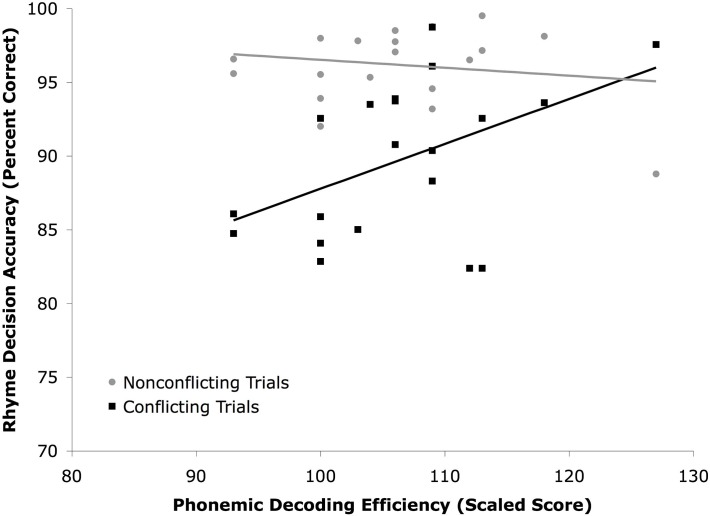
Accuracy (percent correct) of rhyme decisions made on nonconflicting (O+P+ and O-P-) trials and conflicting (O-P+ and O+P-) trials from Experiment 2.

Relationships between PDE score and performance on each type of trial for the rhyme task were explored. PDE scores were not significantly correlated with accuracy for non-conflicting trials, *r*(18) = −.16, *p* > .10, but were correlated with accuracy for conflict trials, *r*(18) = .45, *p* < .05 (Fig. 3). Accuracy was high in the non-conflict condition, regardless of PDE score. However, accuracy in the conflict condition was lower in those with lower PDE scores. A similar pattern was seen with SWE scores, [non-conflict: *r*(18) = −.10, *p* > .10; conflict: *r*(18) = .45, *p* > .05].

## Experiment 2 Discussion

As in Experiment 1, orthographic overlap influenced rhyme judgments and phonological overlap influenced spelling judgments. Participants were slower and less accurate in the face of conflict between orthography and phonology when rhyme and spelling tasks were performed in isolation. Crucially, phonological decoding skill was correlated with the degree to which orthographic conflict impacted rhyme decisions in a second sample. Those participants who demonstrated better reading skills showed a larger impact of orthographic conflict on rhyme decisions.

In Experiment 2, accuracy on trials with orthographic conflict was associated with not just nonword reading skill, but skill at reading familiar words. One possibility is that the relationship with sight word reading observed in Experiment 2 is an artifact of the relatively small sample size and additional data would reveal that no true relationship exists. Alternately, it is possible that presenting the rhyme judgment task in the same session as the spelling judgment task masked relationships between performance on conflicting trials and familiar word reading. From this perspective, perhaps drawing participants’ attention to the spelling of words through the spelling judgment task neutralized the effects of sight word recognition skill on processing conflicting stimuli.

## General Discussion

In two experiments, word spelling was observed to impact a phonological task, in accordance with previous literature [1, 2, 3, 4]. This effect was apparent whether or not it was performed in the same session as a spelling recognition task. For rhyme judgments, participants were both slower and less accurate to accept rhyming pairs when words were spelled differently and to reject non-rhyming pairs when words were spelled similarly. The present work extended this finding to demonstrate that conflicts between orthography and phonology impacted a spelling judgment task. For spelling judgments, participants were more accurate and faster to indicate that word endings were spelled similarly when words shared phonology, and more accurate and faster to indicate that words were spelled differently when phonology differed as well. While effects were apparent at the group level, sizeable individual differences were observed, with some participants showing much larger effects of conflict than others. Notably, in two separate samples, the extent to which orthographic conflict impacted rhyme decisions was associated with reading skill. Individuals with worse reading performance were less accurate in making phonological decisions in the face of orthographic conflict.

While similar effects of orthographic conflict on rhyme decisions have been observed with both visual and auditory word presentations [20, 21], the visual presentation used in the present study may change the interpretation of results. Specifically, visual presentation requires print-to-sound conversion. However, the stimuli chosen for the study were relatively simple, familiar words (monosyllabic, 6 letters or less, high frequency) to lessen the requirement for decoding. The visual presentation may also have served to make the task-irrelevant spelling information more salient in the rhyme condition than it would have been with auditory presentation. Thus, differences in performance may reflect individual differences in the ability to inhibit orthographic processing, rather than individual differences in automatic access to orthography.

In one light, poor phonological decoding ability might reflect weaker links between graphemes and phonemes, and those weak links might allow participants to focus on either orthography or phonology more independently. In the current experiments, the opposite pattern was shown, as worse phonological decoding was associated with greater influence of orthography on phonological decisions. One possible interpretation is that the phonological skills of participants with low PDE scores were particularly weak, and insufficient to support rhyme decisions, while the orthographic skills of those participants were stronger. However, PDE and SWE were significantly associated in both samples. Another possible interpretation is that participants with lower nonword reading scores rely more heavily on orthographic processing or have less ability to inhibit orthographic access while reading than individuals with higher nonword reading scores. While this reliance would often be beneficial, it would be costly when orthography and phonology conflict, as in the word pairs used in this study.

The finding that adults with worse reading performance showed particular difficulty making rhyme decisions for pairs with a conflict between orthographic information and phonological information parallels earlier findings from adolescents with dyslexia [17] and adult literacy students [9]. However, the larger effect of orthographic conflict on poorer decoders conflicts with other studies that have found that younger and poorer readers do not rely more heavily on orthographic information. Studies of younger individuals have tended to reveal that, rather than enhanced sensitivity to orthography, children with dyslexia failed to show effects of orthography [13, 14]. Such a pattern of reduced impact of orthography also characterizes adults whose deficits are persistent or extend beyond phonological decoding. Adults with worse spelling skills showed smaller effects of orthography on an auditory lexical decision task [19]. Adults with dyslexia showed similar effects to typical readers for an auditory rhyme judgment task, and a less pronounced consistency effect for an auditory lexical decision task [11].

One explanation for this apparent disparity is that reliance on orthographic information may characterize mature readers who have somewhat compensated for poor single-word reading skills. Thus, larger effects on orthography might be more apparent in adolescents and adults who struggle to read, but not children, and more apparent among individuals whose educational path might have led a greater degree of compensation (e.g., adolescents, adult literacy students, university students) than adults with dyslexia. The university students in this sample have had years of experience with text, unlike children with dyslexia, and it is possible that those years of experience have shaped their reading systems. Further, the participants in this study all function in a university setting, and are therefore likely to have developed strategies that decrease their reliance on their relatively weak phonological skills.

Therefore, one potential interpretation of the larger impact of orthography among worse readers is that these university students compensate for poor phonological decoding skills with increased reliance on orthography. Previous case studies have provided some support for the idea that some adults may compensate for poor phonological processing with enhanced skill or reliance on orthographic processing. An individual with poor phonological decoding skills excelled at an orthographic choice task, selecting the correct spellings of words more quickly than most control subjects [15]. Another adult with poor phonological skills responded more quickly to irregular words than to regular words, a pattern opposite of that found in control subjects [16]. It is possible that the participants in this study are demonstrating something similar by relying more heavily on orthography in a phonological task. It remains unclear whether this compensatory strategy would be equally apparent on other phonological tasks, particularly those that are not metaphonological in nature.

While effects of orthographic/phonological conflict were observed for both the rhyme and spelling tasks, relationships between the size of the effect and reading skill were apparent only for the rhyme task and were restricted to the measure of nonword reading. This relationship is of particular interest because both measures relate to phonological abilities. The relationships suggests that those adults with poor phonological decoding skills may rely more heavily on orthographic information while making phonological judgments about words, and thus show an enhanced effect of conflict. The lack of relationships with other cognitive abilities suggests that phonological decoding skill specifically, and not intelligence or reading skill more broadly, relates to the effect of orthographic conflict on phonological decisions.

No factors that we measured related to the effect of phonology on the spelling task. However, there was substantial variability across individuals in that effect as well. Thus, while some participants showed greater impact of dissimilar pronunciation on decisions regarding words’ spellings than others, the extent to which an individual was impacted by phonological conflict was not related to nonword reading, sight word reading, or reading comprehension skills. It is possible that variability in the effect of phonological conflict on orthographic decisions would be explained by individual differences in a more sensitive measure of orthographic processing than was used in the present study. While speeding reading of familiar words (the SWE subtest) certainly involves orthographic access, future work might use a more pure measure of orthographic processing skill (e.g., performance on a word-likeness task or measure of sensitivity to bigram frequency). In this context, it is worthy of note that spelling skill has been shown to impact auditory lexical decision performance [19].

This study has limitations. The rhyme task used here is metaphonological, and requires an explicit analysis of phonological information. Therefore, questions have been raised about whether effects observed in this task result from strategy use, rather than reflecting automatic activation of the orthographic code [35]. Whether poor phonological decoders employ different strategies or show different automatic effects, there is nonetheless a relationship between phonological processing ability and the impact of orthography. Additional insight in the locus of the effect would be provided by additional work using other, more natural language tasks. Participants in the study were all adults, and we therefore have limited information regarding their educational background and childhood reading abilities. In order to explore the possibility that enhanced reliance on orthographic processing is a compensatory strategy used by mature readers, longitudinal studies tracking the development of orthographic skills in individuals with poor phonological skills will be necessary.

Overall, this study demonstrated effects of conflict between orthography and phonology on both rhyme and spelling tasks. Individual differences in both the size of these effects and reading skills were observed in a university sample, indicating that some individuals display more sensitivity to this conflict than others. Specifically, individuals with worse phonological decoding skill were more impacted by conflicting orthography in a phonological task. This finding complements prior work, which demonstrated that individuals with strong spelling skills show larger effects of orthography [19], and suggests that variation in the effect of orthographic conflict relates to specific cognitive skills. Thus, poor phonological decoders either display markedly different reading strategies, or show a different degree of automatic access to word form information than individuals with better nonword reading skills. Understanding this variability is important for understanding the reading process more broadly.

## Supporting Information

S1 Appendix Results from Experiment 1 participants who performed the rhyme task first. (DOC)

S1 Table Mean (standard deviation) performance across condition for seven participants dropped from Experiment 1. Accuracies are percent correct and RTs are in ms.
(DOC)

S2 Table Associations (Pearson’s r-values) between effects of conflict and cognitive performance including all participants from Experiment 1. Asterisks indicate relationships which are statistically significant. * *p* < .05, ** *p* < .01
(DOC)

S3 Table Mean (standard deviation) performance across condition for six participants dropped from Experiment 2. Accuracies are percent correct and RTs are in ms.
(DOC)

S4 Table Associations (Pearson’s r-values) between effects of conflict and cognitive performance including all participants from Experiment 2. Asterisks indicate relationships which are statistically significant. * *p* < .05, ** *p* < .01
(DOC)

## References

[1] Seidenberg MS, Tanenhaus MK (1979) Orthographic effects on rhyme monitoring. J Exp Psychol Hum Learn 5: 546–554. 7241059

[2] Ziegler JC, Ferrand L (1998) Orthography shapes the perception of speech: The consistency effect in auditory word recognition. Psychon Bull Rev 5: 683–689.

[3] Kramer AF, Donchin E (1987) Brain potentials as indices of orthographic and phonological interaction during word matching. J Exp Psychol Learn Mem Cogn 13: 76–86. 2949054 10.1037//0278-7393.13.1.76

[4] Rugg MD, Barrett SE (1987) Event-related potentials and the interaction between orthographic and phonological information in a rhyme-judgment task. Brain Lang 32: 336–361. 3690257 10.1016/0093-934x(87)90132-5

[5] Peereman R, Dufour S, Burt JS (2009) Orthographic influences in spoken word recognition: the consistency effect in semantic and gender categorization tasks. Psychon Bull Rev 16:363–368. 10.3758/PBR.16.2.363 19293108

[6] Chéreau C, Gaskell MG, Dumay N (2007) Reading spoken words: Orthographic effects in auditory priming. Cognition 102: 341–360. 16480971 10.1016/j.cognition.2006.01.001

[7] Dijkstra T, Roelofs A, Fieuws S (1995) Orthographic effects on phoneme monitoring. Can J Exp Psychol 49: 264–271. 9183977 10.1037/1196-1961.49.2.264

[8] Ziegler JC, Muneaux M, Grainger J (2003). Neighborhood effects in auditory word recognition: Phonological competition and orthographic facilitation. J Mem Lang 48: 779–793.

[9] Greenberg D, Ehri LC, Perin D (1997) Are word-reading processes the same or different in adult literacy students and third–fifth graders matched for reading level? J Educ Psychol 89: 262–275.

[10] Miller-Shaul S (2005) The characteristics of young and adult dyslexic readers on reading and reading related cognitive tasks as compared to normal readers. Dyslexia 11: 132–151. 15918371 10.1002/dys.290

[11] Pattamadilok C, Nelis A, Kolinsky R (2014) How does reading performance modulate the impact of orthographic knowledge on speech processing? A comparison of normal readers and dyslexic adults. Ann Dyslexia 64: 57–76. 10.1007/s11881-013-0086-8 24352886

[12] Zecker SG (1991) The orthographic code: Developmental trends in reading-disabled and normally-achieving children. Ann Dyslexia 41: 178–192. 10.1007/BF02648085 24233764

[13] Ziegler JC, Muneaux M (2007) Orthographic facilitation and phonological inhibition in spoken word recognition: A developmental study. Psychon Bull Rev 14: 75–80. 17546734 10.3758/bf03194031

[14] Desroches AS, Cone NE, Bolger DJ, Bitan T, Burman DD, Booth JR (2010) Children with reading difficulties show differences in brain regions associated with orthographic processing during spoken language processing. Brain Res 1356: 73–84. 10.1016/j.brainres.2010.07.097 20691675 PMC2942963

[15] Standish VHAJ (1996) Skilled reading with impaired phonology: A case study. Cogn Neuropsychol 13: 1207–1222.

[16] Howard D, Best W (1997) Impaired nonword reading with normal word reading: A case study. J Res Read 20: 55–65.

[17] McPherson WB, Ackerman PT, Holcomb PJ, Dykman RA (1998) Event-related brain potentials elicited during phonological processing differentiate subgroups of reading disabled adolescents. Brain Lang 62: 163–185. 9576820 10.1006/brln.1997.1893

[18] Greenberg D, Ehri LC, Perin D (2002) Do adult literacy students make the same word-reading and spelling errors as children matched for word-reading age? Sci Stud Read 6: 221–243.

[19] Dich N (2011) Individual differences in the size of orthographic effects in spoken word recognition: The role of listeners' orthographic skills. Appl Psycholinguist 32: 169–186.

[20] Kramer AF, Donchin E (1987) Brain potentials as indices of orthographic and phonological interaction during word matching. J Exp Psychol Learn Mem Cogn 13: 76 2949054 10.1037//0278-7393.13.1.76

[21] Rugg MD, Barrett SE (1987) Event-related potentials and the interaction between orthographic and phonological information in a rhyme-judgment task. Brain Lang 32: 336–361. 3690257 10.1016/0093-934x(87)90132-5

[22] Praamstra P, Stegeman DF (1993) Phonological effects on the auditory N400 event-related brain potential. Cogn Brain Res 1: 73–86. 8513242 10.1016/0926-6410(93)90013-u

[23] Grossi G, Coch D, Coffey-Corina S, Holcomb PJ, Neville HJ (2001) Phonological processing in visual rhyming: a developmental ERP study. J Cogn Neurosci 13: 610–625. 11506660 10.1162/089892901750363190

[24] Jackson NE (2005) Are university students’ component reading skills related to their text comprehension and academic achievement? Learn Individ Differ 15: 113–139.

[25] Welcome SE, Leonard CM, Chiarello C (2010) Alternate reading strategies and variable asymmetry of the planum temporale in adult resilient readers. Brain Lang, 113: 73–83. 10.1016/j.bandl.2010.01.003 20223512 PMC4617338

[26] Howard D, Best W (1997) Impaired non-word reading with normal word reading: A case study. J Res Read 20: 55–65.

[27] Holmes VM, Standish JM (1996) Skilled reading with impaired phonology: A case study. Cogn Neuropsychol 13: 1207–1222.

[28] Brown L, Sherbenou R, Johnsen S (1997) Test of nonverbal intelligence: A language- free measure of cognitive ability Austin, TX: Pro-ed.

[29] Torgesen JK, Wagner RK, Rashotte CA (1999) Test of Word Reading Efficiency. Austin, TX: Pro-ed.

[30] Lefly DL, Pennington BF (2000) Reliability and validity of the adult reading history questionnaire. J Learn Disabil 33: 286–296. 15505966 10.1177/002221940003300306

[31] Wilson M (1988) The MRC Psycholinguistic Database: machine readable dictionary, version 2. Behav Res Methods 20: 6–11.

[32] Zeno SM, Ivens SH, Millard RT, Duvvuri R (1995) The educator’s word frequency guide Brewster, NY: Touchstone Applied Science Associates.

[33] Medler DA, Binder JR (2005) MCWord: An on-line orthographic database of the English language. <http://www.neuro.mcw.edu/mcword/>.

[34] Brown JI, Fishco VV, Hanna G (1993) Nelson-Denny Reading Test. Chicago, IL: Riverside.

[35] Damian MF, Bowers JS (2010) Orthographic effects in rhyme monitoring tasks: Are they automatic? Eur J Cogn Psychol 22: 106–116.

