# Supplementary material for: Individual Differences in the Effect of Orthographic/Phonological Conflict on Rhyme and Spelling Decisions
Source: PLoS One. 2015 Mar 9;10(3):e0119734. doi: 10.1371/journal.pone.0119734 (PMC4353721; doi:10.1371/journal.pone.0119734)
Supplement: S1 Appendix — (DOC) [file pone.0119734.s001.doc]

**S3 Appendix.** Results from Experiment 1 participants who performed the rhyme task first.

Eighteen participants in Experiment 1 performed the rhyme task first, and were therefore less likely to be impacted by the presence of the spelling task in the same experimental session. Analyses from this group support analyses from the whole group.

*Overall Effects*

A 2 (task) x 2 (phonology) x 2 (orthography) ANOVA was conducted separately for accuracy and RT. For accuracy, a main effect of task was observed, *F*(1, 17) = 4.92, *p* < .05, with better performance on the rhyme task. The main effect of orthography was not significant (*F* < 1). The main effect of phonology was significant, *F*(1, 17) = 5.08, *p* < .05, with better performance on the non-rhyming items. The interaction of task and phonology was significant, *F*(1, 17) = 27.4, *p* < .001. For the rhyming task, shared phonology led to higher accuracy (90.1% versus 85.6%), while for the spelling task, shared phonology led to less accuracy (77.4% versus 88.1%),). The interaction of task and orthography was also significant, *F*(1, 17) = 17.5, *p* < .005. For the rhyming task, shared orthography led to less accuracy than non-shared (85.1% versus 90.6%), while for the spelling task, shared orthography led to more accuracy than non-shared (86.6% versus 79.0%). Thus, accuracy was higher for the rhyme task when items shared phonology and higher for the spelling task was items shared orthography. Crucially, phonology and orthography showed a significant interaction, *F*(1, 17) = 62.3, *p* < .001. When phonology was shared, shared orthography led to more accuracy (90.9% versus 76.6%). When phonology differed, shared orthography led to less accuracy (80.7% versus 93.0%). The task x phonology x orthography interaction was not significant (*F* < 1.5).

For RT, a main effect of task was observed, *F*(1, 17) = 13.4, *p* < .005, with faster responses to the spelling task. The main effects of phonology and orthography were not significant (*F*s < 1). The interaction of task and phonology was significant, *F*(1, 17) = 68.0, *p* < .001. For the rhyming task, shared phonology led to lower RTs (723 versus 773), while for the spelling task, shared phonology led to longer RTs (707 versus 661). The interaction of task and orthography was also significant, *F*(1, 17) = 69.5, *p* < .001. For the rhyming task, shared orthography led to longer RTs than non-shared (778 versus 717), while for the spelling task, shared orthography led to shorter RTs than non-shared (659 versus 709). Thus, performance was better for the rhyme task when items shared phonology and better for the spelling task was items shared orthography. Crucially, phonology and orthography showed a significant interaction, *F*(1, 17) = 125.8, *p* < .001. When phonology was shared, shared orthography led to faster RTs than differing orthography (656 versus 773). When phonology differed, shared orthography led to longer RTs (780 versus 653). The task x phonology x orthography interaction was not significant (*F* < 3).

For the rhyme task, planned comparisons using paired-samples t-tests indicated that accuracy was higher for O+P+ trials than O-P+ trials [*t*(17) = 2.22, *p* < .05] and higher for O-P- trials than O+P- trials [*t*(17) = 4.55, *p* < .001]. RTs were shorter for O+P+ trials than O-P+ trials [*t*(17) = 5.78, *p* < .001], and shorter for O-P- trials than O+P- trials [*t*(17) = 13.28, *p* < .001]. Thus, orthographic overlap within a trial led to faster and more accurate rhyme decisions and slower and less accurate no-rhyme decisions.

For the spelling task, planned comparisons using paired-samples t-tests indicated that accuracy was higher for O+P+ trials than O+P- trials [*t*(17) = 2.36, *p* < .05] and higher for O-P- trials than O-P+ trials [*t*(17) = 6.24, *p* < .001]. RTs were shorter for O+P+ trials than O+P- trials [*t*(17) = 4.16, *p* < .005], and shorter for O-P- trials than O-P+ trials [*t*(17) = 8.65, *p* < .001]. Thus, phonological overlap within a trial led to better performance when words were spelled similarly worse performance when words were spelled differently.

*Relationships with Reading Skill*

Given that individuals differed in the extent to which orthographic conflict impacted rhyme decisions and phonological conflict impacted spelling decisions, we explored relationships between those individual differences and performance on the standardized measures of nonverbal IQ and reading. Significant relationships emerged between effects of orthographic conflict on rhyme decisions and PDE scores, in both accuracy [*r*(16) = -.49, *p* < .05] and RT [*r*(16) = -.58, *p* < .05]. Specifically, those individuals with better phonological decoding skills were less impacted by orthographic conflict. This relationship was not apparent with Sight Word Efficiency or nonverbal IQ and accuracy (*r*s < .3), but SWE scores were associated with conflict effects in RT [*r*(16) = -.51, *p* < .05]. Additionally, no significant relationships between standardized test performance and spelling effects emerged (*r*s < .4),, suggesting that reading skill relates specifically to the effect of orthographic overlap on phonological judgments, not to effects of conflict more broadly.

Relationships between PDE score and performance on each type of trial for the rhyme task were explored. PDE scores were not significantly correlated with accuracy for non-conflicting trials, *r*(16) = .26, *p* > .10, but were correlated with accuracy for conflict trials, *r*(16) = .47, *p* < .05. Accuracy was high in the non-conflict condition, regardless of PDE score. However, accuracy in the conflict condition was lower in those with lower PDE scores.
