# Supplementary material for: Individual Differences in the Effect of Orthographic/Phonological Conflict on Rhyme and Spelling Decisions
Source: PLoS One. 2015 Mar 9;10(3):e0119734. doi: 10.1371/journal.pone.0119734 (PMC4353721; doi:10.1371/journal.pone.0119734)
Supplement: S1 Table — Accuracies are percent correct and RTs are in ms. (DOC) [file pone.0119734.s002.doc]

**S1 Table.** Mean (standard deviation) performance across condition for seven participants dropped from Experiment 1. Accuracies are percent correct and RTs are in ms.

|  |  | O+P+ | O-P- | O-P+ | O+P- |
| --- | --- | --- | --- | --- | --- |
| Rhyming | Accuracy | 73.6 (33.1) | 87.3 (34.6) | 61.0 (31.0) | 61.3 (34.6) |
|  | RT | 697 (134) | 692 (125) | 759 (142) | 853 (162) |
| Spelling | Accuracy | 82.8 (12.7) | 83.5 (17.6) | 47.6 (30.0) | 61.9 (30.4) |
|  | RT | 701 (158) | 722 (151) | 855 (162) | 734 (151) |
