# Supplementary material for: Individual Differences in the Effect of Orthographic/Phonological Conflict on Rhyme and Spelling Decisions
Source: PLoS One. 2015 Mar 9;10(3):e0119734. doi: 10.1371/journal.pone.0119734 (PMC4353721; doi:10.1371/journal.pone.0119734)
Supplement: S2 Table — Asterisks indicate relationships which are statistically significant. * p < .05, ** p < .01 (DOC) [file pone.0119734.s003.doc]

**S2 Table**. Associations (Pearson’s r-values) between effects of conflict and cognitive performance including all participants from Experiment 1. Asterisks indicate relationships which are statistically significant.

* *p* < .05, ** *p* < .01

|  | Rhyme Accuracy  Effect | Rhyme RT  Effect | Spelling Accuracy  Effect | Spelling RT  Effect |
| --- | --- | --- | --- | --- |
| TONI (Scaled Score) | -.30 | -.05 | .12 | .16 |
| TOWRE SWE (Scaled Score) | -.34* | -.19 | -.03 | .05 |
| TOWRE PDE (Scaled Score) | -.37** | -.34* | -.12 | .01 |
| ARHQ | .42** | .13 | -.22 | -.26 |
