# Supplementary material for: Individual Differences in the Effect of Orthographic/Phonological Conflict on Rhyme and Spelling Decisions
Source: PLoS One. 2015 Mar 9;10(3):e0119734. doi: 10.1371/journal.pone.0119734 (PMC4353721; doi:10.1371/journal.pone.0119734)
Supplement: S3 Table — Accuracies are percent correct and RTs are in ms. (DOC) [file pone.0119734.s004.doc]

**S4 Table.** Mean (standard deviation) performance across condition for six participants dropped from Experiment 2. Accuracies are percent correct and RTs are in ms.

|  |  | O+P+ | O-P- | O-P+ | O+P- |
| --- | --- | --- | --- | --- | --- |
| Rhyming | Accuracy | 74.3 (36.7) | 74.4 (32.7) | 68.5 (23.7) | 41.6 (15.1) |
|  | RT | 703 (104) | 795 (175) | 794 (142) | 939 (215) |
| Spelling | Accuracy | 95.9 | 95.0 | 55.2 | 87 |
|  | RT | 813 | 803 | 909 | 846 |
