# Supplementary material for: Individual Differences in the Effect of Orthographic/Phonological Conflict on Rhyme and Spelling Decisions
Source: PLoS One. 2015 Mar 9;10(3):e0119734. doi: 10.1371/journal.pone.0119734 (PMC4353721; doi:10.1371/journal.pone.0119734)
Supplement: S4 Table — Asterisks indicate relationships which are statistically significant. * p < .05, ** p < .01 (DOC) [file pone.0119734.s005.doc]

**S5 Table.** Associations (Pearson’s r-values) between effects of conflict and cognitive performance including all participants from Experiment 2. Asterisks indicate relationships which are statistically significant.

* *p* < .05, ** *p* < .01

|  | Rhyme Accuracy  Effect | Rhyme RT  Effect | Spelling Accuracy  Effect | Spelling RT  Effect |
| --- | --- | --- | --- | --- |
| Nelson-Denny Comprehension (Scaled Score) | -.48* | -.02 | -.31 | -.25 |
| TOWRE SWE (Scaled Score) | -.51* | .15 | -.14 | -.24 |
| TOWRE PDE (Scaled Score) | -.62** | .15 | .19 | -.39 |
| ARHQ | .36 | -.05 | .09 | -.18 |
